# MicroRNAs in the Host Response to Viral Infections of Veterinary Importance

**DOI:** 10.3389/fvets.2016.00086

**Published:** 2016-10-17

**Authors:** Mohamed Samir, Lea A. I. Vaas, Frank Pessler

**Affiliations:** ^1^TWINCORE, Center for Experimental and Clinical Infection Research, Hannover, Germany; ^2^Department of Zoonoses, Faculty of Veterinary Medicine, Zagazig University, Zagazig, Egypt; ^3^Helmholtz Center for Infection Research, Braunschweig, Germany

**Keywords:** animals, infectious diseases, influenza A virus, miRNAs, viruses, veterinary science

## Abstract

The discovery of small regulatory non-coding RNAs has been an exciting advance in the field of genomics. MicroRNAs (miRNAs) are endogenous RNA molecules, approximately 22 nucleotides in length, that regulate gene expression, mostly at the posttranscriptional level. MiRNA profiling technologies have made it possible to identify and quantify novel miRNAs and to study their regulation and potential roles in disease pathogenesis. Although miRNAs have been extensively investigated in viral infections of humans, their implications in viral diseases affecting animals of veterinary importance are much less understood. The number of annotated miRNAs in different animal species is growing continuously, and novel roles in regulating host–pathogen interactions are being discovered, for instance, miRNA-mediated augmentation of viral transcription and replication. In this review, we present an overview of synthesis and function of miRNAs and an update on the current state of research on host-encoded miRNAs in the genesis of viral infectious diseases in their natural animal host as well as in selected *in vivo* and *in vitro* laboratory models.

## Introduction and Brief History

The recent discovery of non-coding RNAs, such as long non-coding RNAs (lncRNAs) and microRNAs (miRNAs), has begun to direct more and more attention to these potentially very powerful regulatory molecules. The first miRNA was discovered in 1993 during studies of the timing of embryonic development of different larval stages of the worm *Caenorhabditis elegans* (*C. elegans*). In this experiment, Lee and colleagues observed that the RNA transcribed from the *lin-4* locus did not encode a protein but instead silenced the gene encoding Lin-14, an important protein in larval development (1). Since then, the number of studies on miRNAs has been rising rapidly. Indeed, a PubMed search (August 4, 2016; keyword “microRNA”) revealed an increase from 5 entries in 2001 (the first year this term appeared) to 10,189 entries in 2015. MiRNA-encoding genes comprise only 1–5% of the animal genome but have been estimated to affect approximately 30% of all protein-coding genes (2, 3). It is being recognized that their regulatory roles are much more sophisticated than initially thought, owing to the cooperativity (i.e., more than one miRNA species can target the same mRNA) and the multiplicity of their targets (i.e., one miRNA can target hundreds of mRNA species) (4). miRNAs have been shown to play important roles in essentially all biological processes (5), and the differential expression of host miRNAs during infection (1, 6) supports the idea that they may constitute key players in the host response to invading pathogens. We recently summarized trials of therapeutic interventions based on small non-coding RNAs for treatment or prevention of infectious diseases of veterinary importance (7). The current review presents an update on miRNA biogenesis and profiling, discusses some of the challenges encountered when studying them in animals, and summarizes current knowledge of the roles of miRNAs in viral infectious diseases in their respective natural animal hosts. In addition to this, information obtained from cellular and laboratory animal models is presented in cases where data from natural infection are not available or are difficult to obtain. Furthermore, we include some examples of important animal viral diseases where *in vitro* studies have revealed roles for miRNAs. We also discuss miRNA involvement in prion diseases as examples of fatal, untreatable diseases caused by infectious proteins. Viruses, particularly DNA viruses [Marek’s disease virus (MDV), bovine herpesvirus] and even retroviruses (e.g., bovine leukemia virus), can also encode their own miRNAs, but due to space limitations, this topic is not emphasized in this review, and we refer the reader to excellent existing reviews [e.g., Ref. (8, 9)].

## MicroRNA Biogenesis Pathway and Mechanisms of Action

**Figure 1:**
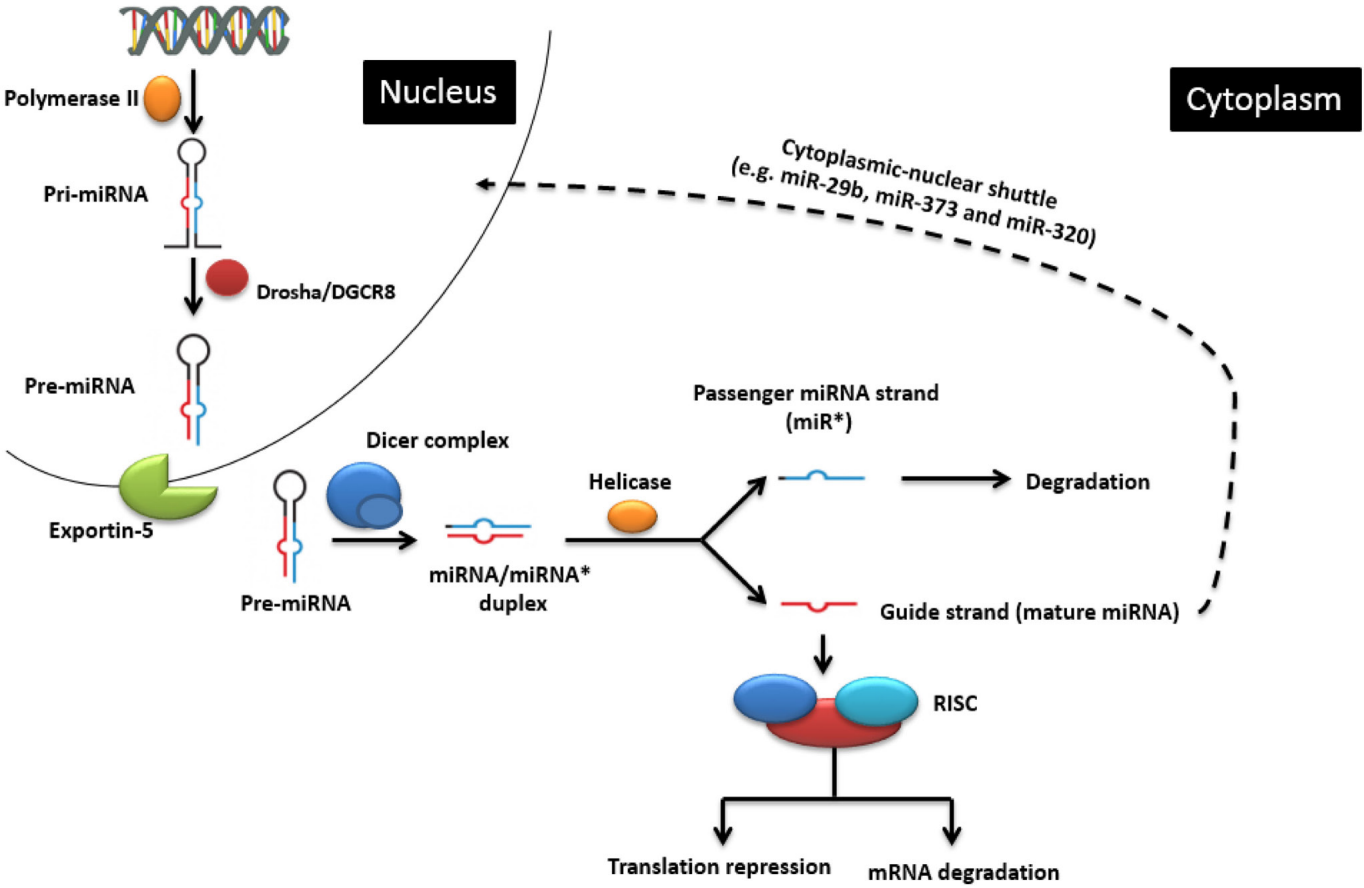
**The classic endogenous miRNA pathway and mechanisms of action**. In the nucleus, pri-miRNA is transcribed by RNA polymerase II and further processed by the Drosha enzyme to produce pre-miRNA, which is then transported into the cytoplasm. There it is cleaved by the Dicer enzyme into the miRNA duplex. The guide strand is uploaded onto the RNA-induced silencing complex (RISC) to regulate gene expression by causing either target mRNA degradation or translation repression. Cytoplasmic-nuclear shuttle is possible for some miRNAs (e.g., miR-29b, miR-320, and miR-373). Adapted from Wikimedia Commons (“Difference DNA RNA-EN.SVG”).

MicroRNAs are non-coding single-stranded oligoribonucleotides of about 22 nucleotides (nts) in length. Their biogenesis and mode of action is illustrated in Figure 1, but the reader is also referred to excellent recent reviews of miRNA biogenesis, e.g., Ref. (10). miRNAs can be transcribed from within protein-coding genes (intragenic miRNAs), from dedicated miRNA coding genes (intergenic miRNAs), or from genes encoding other ncRNA classes, such as small nucleolar RNA (snoRNA) and lncRNAs. More than half of vertebrate miRNA genes lie in introns, implying that most miRNAs are co-expressed with specific host mRNAs (11, 12), although specific transcription start sites for miRNA coding sequences also exist (13). While the majority of miRNA genes are physically separated on the genome (14, 15), many functionally related miRNA genes often, but not always, reside in clusters within the genome (16), probably because they are processed from the same polycistronic transcript (6). When they have their own promoter, miRNA genes are transcribed mainly by RNA polymerase II and, rarely, by RNA polymerase III (17) to form primary miRNAs (pri-miRNAs) (18), which are then folded to produce hairpin structures. Each hairpin structure consists of a 32-nt-long imperfect stem and a large terminal loop. The enzyme Drosha and its cofactor DiGeorge syndrome critical region 8 (DGCR8) cleave the 22 nts downstream of the stem to yield 60-nt-long precursor miRNAs (pre-miRNA) (18). The pre-miRNAs are then exported to the cytoplasm *via* exportin 5 where the terminal loops are excised by Dicer and tar RNA binding protein (TRBP) to produce short imperfect miRNA duplex intermediates (19). This duplex is then unwound by a helicase into two miRNA strands. One strand (the 5′-strand or guide strand for most miRNAs) is incorporated into the RNA-induced silencing complex (RISC) to target mRNAs, and the other strand (3′-strand, also referred to as miRNA*) is usually degraded but can also persist and take on regulatory roles of its own.

Apart from the classic biogenesis pathway, Dicer or AGO-2-independent (non-canonical) pathways have also been described (20). For instance, mirtrons are miRNAs that are produced from introns by the splicing machinery instead of the Drosha processing complex (21). The maturation of some miRNAs (for example, miR-451) is Dicer independent, but AGO dependent. This is possibly because the stem part of its pre-miRNA is too short (17 bp) to be processed by Dicer (22).

MicroRNAs mainly act in the cytoplasm, namely, by repressing mRNA translation and, to a lesser extent, by inducing mRNA degradation. Actually, the two processes can be connected in that decreased translation may lead to loss of stability. Usually, the seed region (seven or eight consecutive nts at the miRNA 5′-end) binds to complementary sequences in the 3′-untranslated region (UTR) of the target mRNA (23), but miRNAs can also target other sequences, such as the 5′-UTR or coding sequences (24). Remarkably, some miRNAs have been implicated in actually stimulating translation (25, 26). miRNAs can also affect gene expression at the level of pre-mRNA splicing. Usually, this occurs indirectly in that a miRNA regulates translation of a protein involved in differential splicing. miR-124 represents a good example of this: during neuronal differentiation of the mouse, it binds to the mRNA encoding a repressor of alternative splicing, PTBP1, leading to increased synthesis of the PTBP2 protein. The latter, in turn, induces alternative splicing programs leading to neuronal differentiation (27).

Interestingly, mature miRNAs can also be found in the nucleus (28–30). Profiling studies of fractionated cells revealed the enrichment of miRNAs, including miR-320, miR-373, and miR-29b, in the nucleus of cell lines derived from various origins (28, 29, 31–34). The driving force of this cytoplasmic-nuclear shuttle, at least for miR-29b, is thought to be a miRNA-associated hexanucleotide terminal motif (AGUGUU) which increases the stability of the miRNA (29). Their localization in the nucleus suggests that these nuclear miRNAs affect gene expression at critical steps that take place in the nucleus, notably transcription. One mechanism by which they can affect transcription is to promote gene silencing by inducing epigenetic changes, such as histone modifications or methylation of promoter elements (35, 36). Remarkably, it has been shown that a nuclear miRNA can even stimulate transcription by binding to a complementary promoter sequence (34). Taken together, these studies suggest that the scope of miRNA functions has become broader than previously thought. miRNAs themselves can be regulated by several mechanisms. In general, miRNA half-life ranges from hours to days, but this obviously varies depending on the organ, body fluid, and cell type (37). Compared to other RNA species, their stability in stored biosamples is generally much higher than that of other RNA species, which increases their value for biomarker studies using biobanked animal or human biosamples (7). For instance, in contrast to mRNA, they are highly stable in formalin-fixed paraffin-embedded tissue blocks, allowing for localization and expression studies even after years of storage (38). Active degradation can be mediated by small RNA degrading nucleases (SDNs) [reviewed in Ref. (39)]. miRNAs can also be extruded from the cell into body fluids (e.g., blood, CSF, urine) in exosomes or microvesicles. On one hand, this may be an additional mechanism by which intracellular miRNA populations can be regulated. On the other hand, these vesicles can also function as vehicles by which miRNA can spread systemically and potentially be taken up by accessible cells (40, 41). More recently, it was found that miRNA activity can be inhibited by sequestration by circular RNAs (“miRNA sponges”) (42).

## Update on Identification of miRNAs and Computational Prediction of Their Targets in Animals of Veterinary Importance

**Figure 2:**
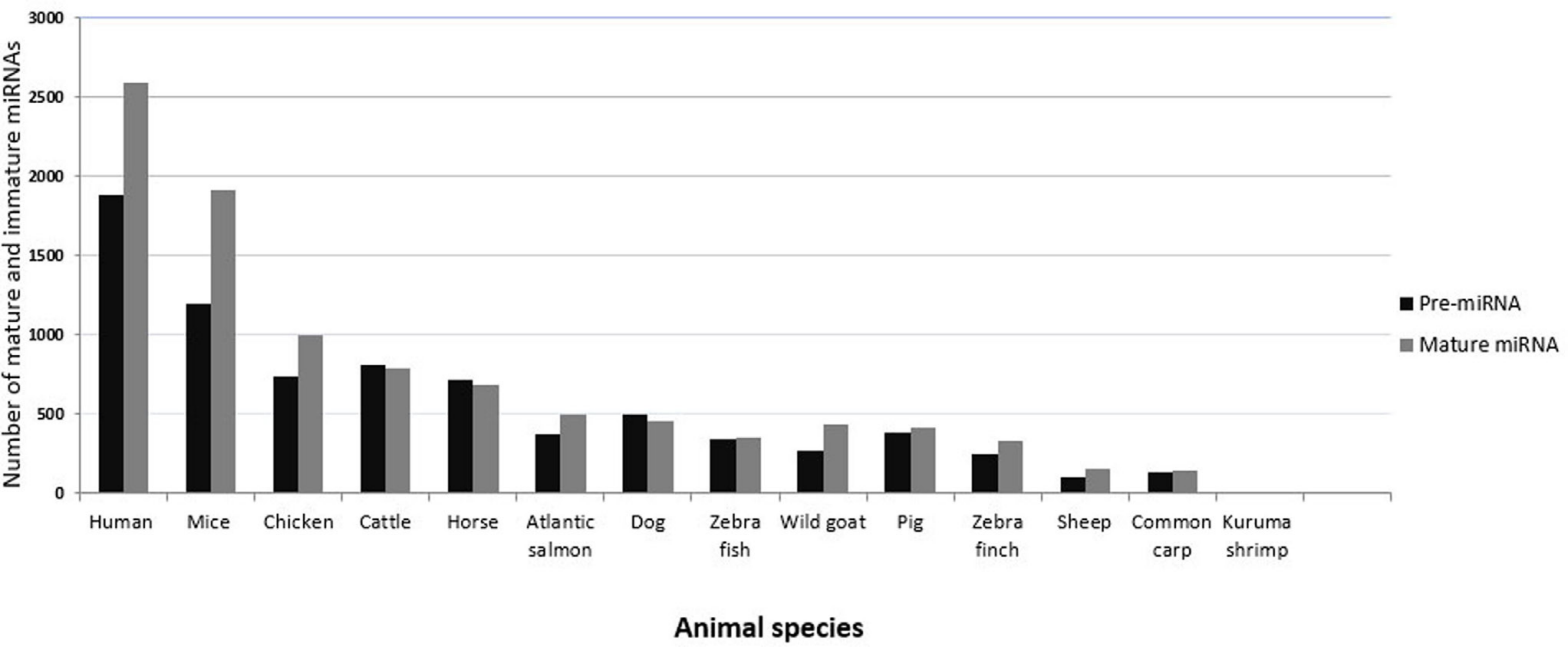
**Numbers of currently annotated mature and immature miRNAs in selected species**. The most recent numbers of immature (black bars) and mature (gray bars) miRNAs in animals of veterinary importance are plotted on the *y*-axis. Values for humans and mice are shown for comparison. Data were obtained from miRBase version 21.

In vertebrates, miRNAs have been studied most extensively in humans and mice, in part because much fewer miRNAs have been annotated or made publicly available in other organisms, including animals of veterinary importance (43). Figure 2 shows the number of currently annotated mature and immature miRNAs in different animal species of veterinary importance, as well as in humans and mice, according to miRBase version 21 (43). Humans and mice had the highest number of annotated miRNAs, followed by chicken, cattle, and horse. miRNA expression profiling has assisted in identifying miRNAs that regulate a range of biological processes, and there are several established and emerging methods for measuring miRNA expression profiles in biological samples. The commonly used approaches include reverse transcription quantitative real-time PCR (RT-qPCR), microarrays, and RNA sequencing (RNA seq) (44, 45). In the process of discovering novel miRNAs, the analysis of profiling data follows the same principle in that the generated reads have to be mapped to the reference genome of the concerned species. In this context, the lack of a published reference genome always represents a limitation. The unique feature of cross-species conservation of miRNAs has aided in the identification of miRNAs or their targets especially in the species where the genome (or the miRNome) is not completely annotated (46). The miRNAs from such species have been identified by homology searches where the deep sequencing reads are aligned against the genome of the most closely related species (47). Indeed, most bovine miRNAs have been identified in this way, and a similar method was used to identify goat and sheep miRNAs (48). Researchers from China recently characterized miRNA species in skin and ovaries of ducks (49, 50). An alternative strategy was used in these studies. First, the reads were filtered and then mapped by blast alignment to all known mature chicken miRNA sequences present in miRBase. The sequences that did not correspond to chicken miRNAs were then mapped to miRNAs in other species. This strategy has also been applied to the Chinese hamster (51). There are several computational resources for the identification of miRNAs and their targets (52). Among these, miReader is a newly launched bioinformatics tool for the discovery of novel miRNAs that can be used to identify miRNAs that are not annotated in miRBase, yet without the need for reference genomic sequences or homologous genomes. It shows a high degree of accuracy in a wide range of animal species (47). The prediction and validation of miRNA targets are essential steps in determining their regulatory function. In this regard, the imperfect complementarity between the miRNAs and their mRNA targets represents a major challenge because of potentially false positive predictions. Nevertheless, various online target prediction tools have been developed. Prominent tools include TargetScan (53), miRanda (54), PITA (55), and RNA hybrid (56). Most of these tools rely on basic principles, such as miRNA/mRNA pairing, cross-species conservation of the mRNA 3′-UTR, and the free energy required to form the duplex (57, 58). Kiriakidou indicated that the agreement of mRNA targets predicted for a set of 79 miRNAs by several target prediction tools was only in the range of 10–50% (59). One reason for this may be the divergent use of the degree of conservation. For example, conservation may be used but not directly incorporated into the score (as in PITA), or not used at all (as in RNA22) (60). It is worth mentioning that these tools exhibit some differences, which might be independent of the algorithms, such as using different UTR databases for prediction, cross-species comparison, or alignment artifacts. Other among-tools variations that are related to the prediction algorithms themselves include the number of nts involved in pairing (canonical, marginal, and atypical seed-matched sites) (61), the method used to measure 3′-UTR conservation, the accessibility to the UTR, and the statistical approach used (62). The computational approaches for predicting miRNA–mRNA binding revealed wide variation and bias. Therefore, various experimental strategies have been developed in humans, for instance, “Cross-linking and sequencing of hybrids” (63), which allow the identification of miRNA–target pair chimeras in deep sequencing data (64). Some attempts have been successful to use the mRNA targets to predict biological pathways that are regulated by a set of miRNAs. For instance, the DNA intelligent analysis (DIANA) tool (65) offers the miRPath server, which can create hierarchical clusters of miRNAs and pathways based on the levels of the predicted miRNA/mRNA interactions. Generally, a major limitation of the available web tools is that they include a limited number of species (e.g., human, mouse, *C. elegans*), leaving many animal species underrepresented, in particular those of veterinary importance. However, some tools (e.g., TargetScan, miRDP, and Microcosm) include some species of veterinary importance, such as chicken, dog, and cow (3, 66, 67). Oasis is a comprehensive online tool for the analysis of RNAseq data that allows for target prediction and analysis of novel (putative) miRNAs, including prediction of miRNomes of species without annotated miRNomes (68). The growing list of annotated miRNAs in species of veterinary importance undoubtedly warrants better inclusion of such species in the online prediction algorithms. Considering the probability of obtaining false positive and negative results using the online prediction algorithms, it seems critical to confirm miRNA function using experimental work. The function of a miRNA can be validated by several experimental approaches. The luciferase reporter assay can be used to test for binding of a miRNA to a predicted target sequence (69). To detect the effect of miRNA–mRNA pairing on the protein level, immunoblotting can be employed. For a detailed analysis of procedures used for the experimental validation of miRNA targets, we recommend references (70–72). *In situ* hybridization using a labeled antisense probe can be used to investigate which cell type in a tissue expresses a certain miRNA (73), but this is technically not easy, for instance, because an endogenous counterpart miRNA that also contains the antisense sequence may compete with binding of the probe to the target miRNA.

## MicroRNAs in the Host Response to Viral Infections of Veterinary Importance

Considering the implication of miRNAs in nearly all biological processes, links between miRNAs and disease status are expected. Earlier reports suggested that miRNAs are involved in the regulation of inflammatory pathways as well as adaptive and innate immunity, stress factors, and cytokine signaling (74). Diseased tissues may show unique miRNA expression patterns, which subsequently might affect virus replication and/or survival. For instance, miRNAs might promote virus replication by direct pairing with virus-derived transcripts, as has been shown for miR-122 and hepatitis C virus (75). A similar mechanism is utilized by bovine viral diarrhea and classic swine fever viruses, where miR-17 and let-7 were found to bind their 3′UTRs, thus enhancing stability and translation of viral mRNA (76). miRNAs may restrict viral replication, as exemplified by miR-32 in primate foamy virus type 1 infection (77).

When studying regulation of host- or virus-encoded RNA targets by miRNAs, the “abundance problem” needs to be considered. Based on studies in cell lines, it has been argued that approximately 100 copies of a miRNA are needed to affect host transcripts and, depending on the number and activity of viral genomes in the cell, likely a higher number to affect viral transcripts (78). Thus, expression changes of low copy number miRNAs during infection may be of little functional consequences. Studies on regulation of host miRNAs in infection often ignore this potentially important issue.

Studies of changes in miRNA populations in infections in the natural host are clearly limited by the fact that they usually do not allow to distinguish between cause and effect (and therefore do not allow to draw conclusions as to mechanisms) but are mostly suitable for biomarker studies and for formulating hypotheses based on the descriptive data obtained. These can then be tested in the appropriate experimental models, if available. Another point to be considered is that a good part of the host miRNome response to infection may be due to collateral cell and tissue damage and turnover and not due to the immune response *per se*. Clearly, our understanding of the mechanistic associations and implications of miRNAs in animal viral infectious diseases is still far from complete. Here, we review the literature that describes the expression of miRNAs in the context of viral infectious diseases that affect farm and pet animals, with special emphasis on infections in the natural host. Additionally, we discuss miRNA expression in infectious viral diseases in laboratory *in vivo* and *in vitro* models where there are no sufficient data involving the natural host.

### Infections in the Natural Host

#### Influenza A Virus

Infection with influenza A virus (IAV) has a negative impact on the poultry and swine industries, and on human health in that it is able to cross species barriers and adapt to the human host. The differences in pathogenesis of various IAV strains are attributed to both viral and host factors. Yet, there remains an urgent need for diagnostic markers to sense the very early phases of IAV outbreaks at the farm level. The first trial to emphasize the effect of miRNAs on IAV pathogenesis in a species of veterinary importance attempted to define miRNA populations in lung and trachea of commercial Leghorn chickens experimentally infected with H5N3 virus (79) (Table 1). Some miRNAs were upregulated in both lung and trachea, and others showed a tissue-specific pattern. For instance, miRNA-206 was more highly expressed in infected than in non-infected lungs, while the reverse was reported for trachea. These findings suggest that specific host miRNA regulatory mechanisms might exist in response to IAV infection (79). The same research group subsequently confirmed that host cellular miRNAs following H5N3 virus challenge could lead to different results depending on the host genetic background (80). They proved that host-encoded miRNAs were modulated differentially in the lungs of broiler and layer chickens (Table S1 in Supplementary Material). In broilers, more miRNAs were upregulated than downregulated, whereas this was reversed in layers. Only two miRNAs, miR-1599 and miR-1416, were consistently regulated independent of chicken breed. Since this study showed a breed-dependent effect on miRNA expression, the authors proposed that miRNA expression is linked to immunity. Some of the identified miRNAs have predicted target sites in immune-related genes, such as *IL17RD, ARL11, CHMP2B, POU1F1, PDHB*, and *HIF1AN*. Indeed, broiler chickens have weak short-term humoral immunity, whereas layers possess a long-term humoral immune response and strong cellular immunity, which goes in line with the fact that layers have a longer life expectancy (81). Regarding immunity-related miRNAs, Li and his colleagues claimed that miRNAs account for part of the immune-related differences between chickens and ducks upon H5N1 infection. They showed that, in contrast to duck, the dynamics of the miRNA repertoires of chicken spleen, thymus, and bursa changed upon infection, with more miRNAs being upregulated than downregulated (Table S1 in Supplementary Material). A set of spleen-specific miRNAs were found to target genes in the B-cell receptor pathway (82). This study potentially explains the differential susceptibility to IAV infection between chickens and ducks based on miRNA expression differences. In pig, miRNAs were found to be dysregulated in the lungs after aerosol challenge with reassortant IAV (H1N2). Some miRNAs were upregulated 1, 3, and 14 days after infection (miR-15a); others were expressed late (miR-21, miR-206, and miR-451) or transiently upregulated (miR-223), whereas miR-146 was transiently downregulated (Table 1). These miRNAs target several inflammation-related molecules (83). In a recent experiment that also involved pig, Jiang et al. showed that miRNAs of piglet pulmonary alveolar macrophages differed in expression during acute (4 days post infection) and recovery (7 days post infection) phases of IAV (H1N1) infection. By day 4, most of the miRNAs (*n* = 70) were downregulated, presumably allowing an increase in their target mRNAs that participate in the host defense against viruses. Three days later, the expression levels of most miRNAs returned to normal, with subsequent normal expression of immune genes during the recovery phase (Table S1 in Supplementary Material) (84). The isolation of H3N8 in 2005 from infected dogs in the United States, and the identification of H3N2 in 2007 from dogs in Korea and China marked the emergence of canine influenza virus (85, 86). In 2014, Zhao et al. conducted an experiment in which they profiled the miRNA expression patterns in lung and trachea of beagles experimentally infected with H3N2 virus (87). In this study, 34 and 45 miRNAs were differentially expressed between infected and non-infected groups in lung and trachea, respectively (Table S1 in Supplementary Material). In addition, 99 miRNAs were differentially expressed between infected lung and trachea. Interestingly, miRNA expression levels were higher in infected than in non-infected lungs, the reverse was reported in trachea, indicating a tissue-specific signature of miRNAs and suggesting that these miRNAs may play different roles in different organs. The divergence in the results obtained from various studies might be due to different strains or models used. Other publications have documented miRNA involvement in the host response to IAV infection in animals and humans, but using cell-based models (88–91). Considered together, these studies suggest that a specific host miRNA response is associated with IAV infection and could contribute to the pathogenesis of IAV including its tissue/cell tropism and host preference.

**Table 1:** **Selected studies reporting differentially expressed host-encoded miRNAs in animal viral infectious diseases of veterinary importance (*in vivo* studies of natural infection and laboratory models)**.

Disease reservoir	Disease or pathogen	miRNAs		Function(s)	Tissue	Reference
		Upregulated	Downregulated			
Horse	Venezuelan equine encephalitis virus (VEEV)	Various (*n* = 24)^a^	Various (*n* = 43)	Cell death, apoptosis, and inflammation	Brain	(92)
Layer chickens	Influenza A virus (H5N3)	miR-1a, miR-140, miR-449	miR-181a	Regulation of host and viral gene expression	Lung and trachea	(79)
Layer chickens	Influenza A virus (H5N3)	miR-445, miR-34b, miR-34c, miR-1a-1, miR-1a-2, miR-1b, miR-449, miR-140	miR-181a	Regulation of host and viral gene expression	Trachea	(79)
Broiler chickens	Influenza A virus (H5N3)	Various (*n* = 25)^a^	miR-449b, miR-460a, miR-206, miR-301, and miR-187	Regulation of host and viral gene expression	Lung	(80)
Dog and carnivore	Rabies	miR-1894-5p, miR-290-3p, miR-1901, miR-207, miR-1896, miR-715, miR-3470b, miR-146b^a^, miR-203, miR-770-5p	miR-200a, miR-200b, miR-200c, miR-182, miR-183, and miR-429	Targeting RIG-1 like receptor, RIF3 (a target for miR-203), and TRIM25 (a target of miR-207)	Brain	(93)
Pig	Influenza A virus (H1N2)	miR-21, miR-15a, miR-206, miR-451, miR-223	miR-146	Inflammation	Lung	(83)
Cattle, sheep, goat, cat	Prion protein-related diseases	miR-342-3p, miR-320, let-7b, miR-328, miR-128, miR-139-5p, miR-146a	miR-338-3p and miR-337-3p	Regulation of pro-inflammatory cytokine production	Brain	(94, 95)

*The column “Tissue” refers to the tissue in which the respective profiling study was conducted. Expression in human tissues is reported in Ref. (96). The column “Function” refers to the function of all miRNAs contained in the corresponding column. Due to space limitations, several other studies are contained in Table S1 in Supplementary Material*.

*^a^A complete list of all miRNAs is provided in Table S1 in Supplementary Material*.

##### Influenza A Virus Infection as an Example of Cross-Species Conservation of Host-Encoded miRNAs

**Figure 3:**
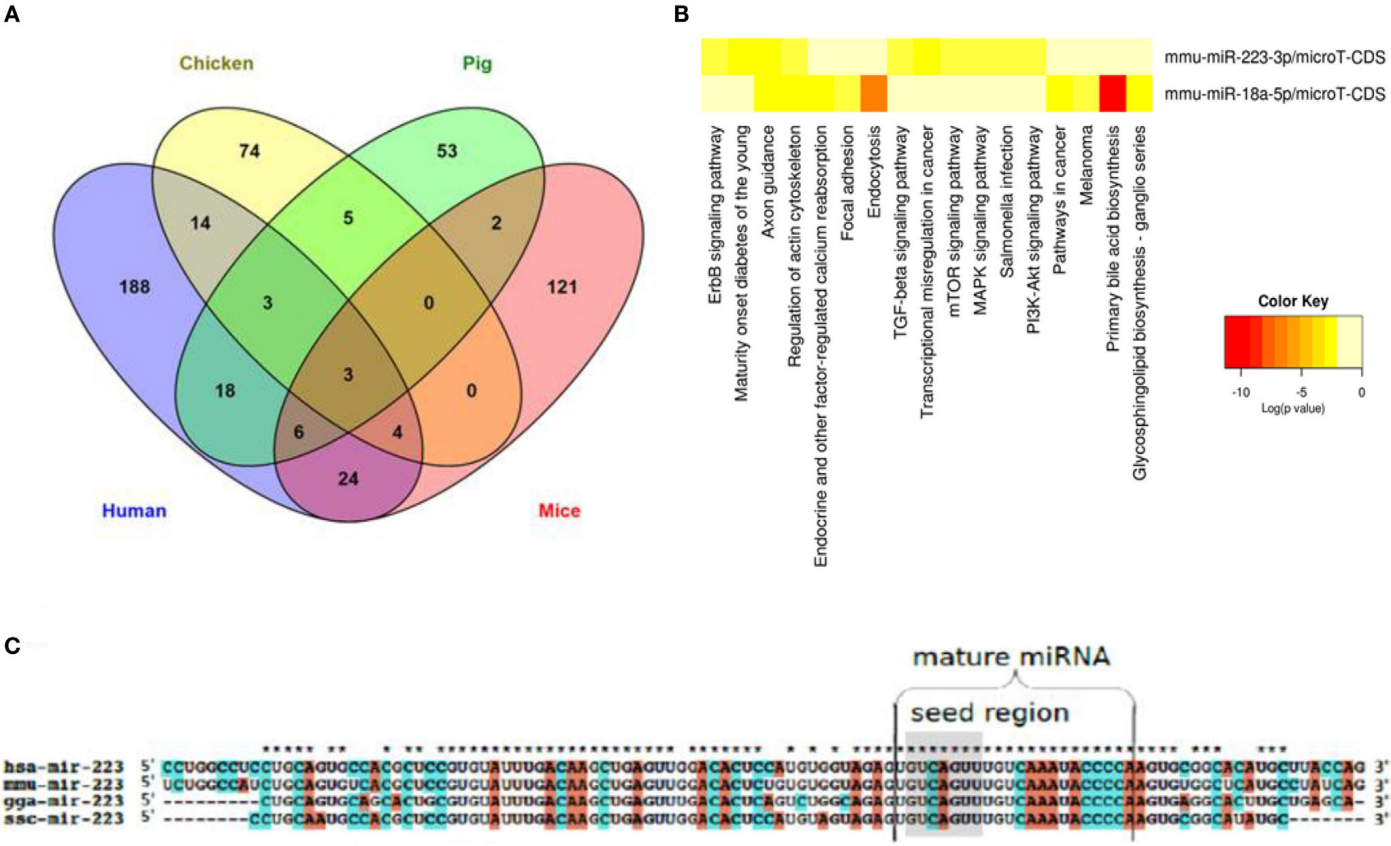
**miRNAs that are regulated in IAV infection in humans, chicken, pig, and mice**. **(A)** Venn diagram depicting miRNAs that are differentially and commonly expressed in the four species. **(B)** Heat map showing the pathways regulated by two of the three miRNAs regulated in all four species. **(C)** Sequence alignment of the premature form of miR-223, a well characterized miRNA that is regulated in all four species. While the mature form of this miRNA is highly conserved across the four species (represented by stars above the sequences), the premature form shows a lesser degree of conservation. The seed region is highlighted in gray.

All IAV subtypes primarily originated in wild birds that are classified under the orders Anseriformes and Charadriiformes (97). Their migration and aquatic nature enable both the maintenance of IAV strains as well as the emergence of novel strains in spillover hosts. While mice can be infected with IAV only after serial passages (98), domestic chicken, swine, and humans are among the main transmission reservoirs (99). Species-dependent variation in the host response to IAV has been reported, including in chickens and ducks (100). Despite their potential roles in interspecies differences in host responses to infections, a global view of cross-species expression, conservation, and functionality of miRNAs is incomplete and spread across several studies. In order to obtain an overview of shared and distinct miRNAs in an infectious disease that affects both animals of veterinary importance and humans, we compiled the literature on miRNAs regulated upon IAV infection in humans (91, 101, 102), mice (103–108), chicken (79, 80, 82), and pig (83, 84), extracted all miRNAs (separately for each species) that have been shown to be differentially regulated upon IAV infection (Table S2 in Supplementary Material), and selected those miRNAs that are regulated in all four species. Naturally, this analysis needs to be interpreted knowing that its negative predictive value is low due to publication bias and differences in the numbers of studies done in the various hosts; on the other hand, the positive predictive value likely is high. As shown in Figure 3A, three miRNAs (miR-18a-5p, miR-223-3p, and miR-451-3p) were differentially regulated in all four species, suggesting a common IAV infection-related signature. Limiting the analysis to the three natural hosts (humans, chicken, and pig) revealed an additional three consistently regulated miRNAs (miR-18b-3p, miR-22-5p and miR-30a-3p). The expression patterns and tissue specificities of these miRNAs are listed in Table 1 and Table S1 in Supplementary Material. These six miRNAs are related to diverse biological processes. The role of miR-223 in infection, inflammation, and cancer has been reviewed extensively (109). An association between miR-223 and IAV virulence has been proposed in several studies. Among these, Li et al. observed an upregulation of miR-223 in mice infected with the lethal r1918 pandemic H1N1, but not the less virulent Tx/91 strain. The authors also reported that miR-223 can indirectly repress the transcription factor CREB, which is responsible for maintaining cell survival and growth (110), suggesting that the lethal IAVs may induce cellular apoptosis *via* increasing expression of miR-223 (103). Using a luciferase-based reporter assay, Haneklaus et al. showed that overexpression of miR-223 tends to reduce luciferase expression in the vector containing the 3′-UTR of NOD-like receptor P3 (NLRP3), implying that miR-223 can target NLRP3 with subsequent reduction in activity of the NLRP3-inflammasome (111). In another study, miR-223 was shown to be upregulated in lung of mice experimentally infected with two IAV (H5N2) strains of differential virulence. The more virulent strain induced miR-223 more strongly than the less virulent strain, and subsequent administration of anti-miR-223 to these mice reduced IAV titer and increased animal survival and weight gain at 1, 3, and 5 days after inoculation, suggesting a positive correlation between miR-223 levels and severity of IAV infection in this model (112). IAV infection leads to changes in expression of miR-155 in humans, chickens and mice, but in pigs, interestingly, only of its counterpart miR-155-3p. MiR-155 has been found to regulate both innate and adaptive arms of the immune system. On the one hand, CD^8+^ T cells transduced with the MigR1 vector overexpressing miR-155 showed a higher expansion and proliferation rate compared to the negative controls, suggesting a role for this miRNA in the T cell response (113). On the other hand, overexpressing and blocking miR-155 in a murine macrophage cell line indicated that miR-155 can target the suppressor of cytokine signaling (SOCS), a negative regulator of IFN-α, thereby triggering the antiviral type 1 IFN response (114). In support of this, Choi et al. found that mice injected with anti-miR-155-3p displayed a dramatic loss in body weight and succumbed to IAV infection within 8 dpi with high viral titers in their lungs (112). These studies suggest that IAV may usurp host miRNAs for its own benefits. The pathogenesis of IAV involves effector molecules that converge to form interacting signaling pathways (115). To test the hypothesis that there are common miRNA-regulated functional pathways that are induced upon IAV infection, we utilized the bioinformatics tool DIANA miRPath v.2.0 (65) to predict pathways that might be regulated by these miRNAs. For this purpose, we chose the smaller set of miRNAs regulated also in the mouse (an adaptive, well-studied host for IAV) and the murine functions of the DIANA tool, as it does not support analyses of chicken and pig miRNAs, and also because the mouse is the one of the four species with the best experimental evidence for miRNA function at the organismal level. Figure 3B shows the pathways regulated by two of these three miRNAs, i.e., miR-18a-5p and miR-223-3p (miR-451-3p is not included in the DIANA tool). Among the identified pathways, endocytosis and the PI3K–Akt, MAP kinase, mTOR, and TGF-β signaling pathways are known to be associated with IAV–host interaction. The PI3K pathway is mainly involved in apoptosis. Binding of the NS1 gene of IAV to the P85β regulatory subunit activates PI3K, leading to phosphorylation of the downstream effector molecule Akt, which further phosphorylates both caspase 9 and GSK-3β, thereby suppressing apoptosis and prolonging virus infection (116). Hirata et al. suggested that inhibiting Akt kinase activity may have therapeutic advantages for IAV infection by interfering with viral entry and replication (117). Besides the PI3K pathway, MAP kinases have been reported to promote IAV ribonucleoprotein capsid trafficking and virus production (118) and to regulate the production of RANTES, a chemokine that is released by lung epithelial cells and alveolar macrophages (119). Among the identified pathways, the TGF-β signaling pathway is known to be activated by the IAV neuraminidase. The authors claimed that the modulation of TGF-β activity during IAV infection influences viral titers and disease outcome in experimentally infected mice, suggesting a significant role for TGF-β signaling in IAV pathogenesis. Interestingly, this study postulated that the inability of H5N1 virus to trigger the TGF-β cascade might explain the improper host immune response and the exaggerated immune pathology seen in many H5N1 cases (120). Clathrin-mediated endocytosis is a prerequisite for IAV entrance into target cells (121). For further descriptions of the implication of the predicted IAV-associated pathways, we recommend references (115, 122). The remaining 12 pathways are mainly related to cancer, but we cannot rule out any possible roles for these pathways in IAV infection, as cancer-related pathways are often also involved in the host response to infection. To gain further insight into the cross-species conservation of functionally related miRNAs, we then used the ClustalX version 2.0 software (123) to align the sequences of the stem-loop (premature) form of miR-223 in humans, mice, chicken, and pig (Figure 3C). The degree of conservation was higher in the mature miRNA than in the rest of the stem-loop sequence. In terms of sequence identity, the mature form of miR-223 was more closely related to the corresponding sequences in mice than in chicken and pig. The seed region was 100% identical among the four species studied. Shared seed sequences might indicate shared miRNA targets, lending further support to the notion that these miRNAs play similar roles in these species in the host response to IAV infection. Additional functional implications of these shared miRNAs in the course of IAV infection are discussed in Ref. (7).

#### Infectious Bursal Disease Virus

Infectious bursal disease virus (IBDV) is the etiologic agent of infectious bursal disease, which is a highly contagious disease that predominantly affects the bursa of Fabricius in birds (124). Although vaccination has contributed to the overall reduction of disease burden in poultry (125), several challenges remain to be overcome, and new approaches are needed to fight IBDV (126). The initial study that indicated an anti-IBDV effect of miRNAs in its natural host was conducted in 2011 (127). In this experiment, the authors reported that transducing 8-day-old SPF chicken embryos with an adeno-associated virus vector carrying VP1- and VP2-specific miRNAs resulted in reduced replication of the Lukert strain of IBDV by 48 hpi. In a subsequent study, gga-miR-9* was found to be induced 2, 4, 12, and 24 h after infection with IBDV (128). The authors also provided evidence that miR-9* can promote IBDV replication by repressing the production of IFN. These data indicate that miRNAs can either stimulate or inhibit BDV infection and that their roles as therapeutic interventions merit further investigations.

#### Marek’s Disease Virus

Marek’s disease is a highly contagious viral neoplastic disease of chicken that results from infection with Gallid herpesvirus 2 (GaHV-2), also known as MDV (129). The disease has remained a major concern in poultry owing to the continual emergence of new virulent strains (130). Although several *in vitro* approaches have been employed to unravel the roles played by host-encoded miRNAs in different scenarios of MDV infection (131–136), fewer studies have addressed their role in the natural host. Initially, deep sequencing of samples from MDV-infected chicken spleen and liver revealed that 187 miRNAs were differential expressed compared to the non-infected tissues. These miRNAs were found to target genes that are related to lymphomagenesis (134) (Table S1 in Supplementary Material). Stik et al. (137) used white leghorn chicken experimentally inoculated with the oncogenic RB-1B strain as a model to investigate connections between the chicken miRNA response and the oncogenic nature of MDV. This study reported that gga-miR-21 was upregulated in chicken inoculated with the oncogenic strain, as compared to chicken vaccinated with the non-oncogenic strain CVI988 and non-infected chicken. gga-miR-21 was also found to promote tumor formation by targeting the chicken programmed cell death 4 (PDCD4) mRNA. In the same context, miR-103 was found to be downregulated in tumor samples from spleen and liver of infected chickens, where it targets cyclin E1 and the transcription factor Dp-2, which normally causes suppression of cell migration and tumor formation (138). Similarly, miR-26a was found to be downregulated in MDV-infected chicken spleens during different phases of tumor formation (139). This miRNA targets the “Never In Mitosis Gene A-related kinase 6” gene, which was strongly upregulated in the same samples (suggesting loss of inhibition due to miR-26a) and has been linked to cell proliferation. Collectively, these *in vivo* studies emphasized the role of host miRNAs in the development of MDV-associated tumors in chickens. In terms of susceptibility to infection, Tian et al. identified 64 miRNAs (58 downregulated and 6 upregulated) that were differentially expressed between MDV-infected and non-infected chicken cells when the susceptible line 7_2_ was used, but not when the resistant cell line 6_3_ was used (140). The differentially expressed miRNAs were found to target components of several immune-related pathways, such as NF-κB signaling and T-cell and B-cell receptor signaling, raising the possibility that miRNAs might influence the genetic susceptibility of chicken to MDV infection through controlling immune responses.

#### Avian Leukosis Virus

Avian leukosis virus (ALV) belongs to the genus *Alpharetrovirus* of the *Retroviridae* family. This virus is capable of inducing tumors in avian hosts, including B-cell lymphoma, hemangioma, and myelocytoma (141). As discussed above in MDV infection, miRNAs have proven to be strongly associated with virus-induced avian tumors through regulating cell proliferation or cell death (141, 142). Among the first studies on miRNA-mediated control of ALV-J infection is the one conducted by Li et al. in 2012. They investigated miRNA expression in the liver of ALV-J-infected 10-week-old chickens. They proposed that seven upregulated miRNAs (gga-mir-221, gga-mir-222, gga-mir-1456, gga-mir-1704, gga-mir-1777, gga-mir-1790, and gga-mir-2127) might play a tumorigenic role, whereas downregulation of five other miRNAs (gga-let-7b, gga-let-7i, gga-mir-125b, gga-mir-375, and gga-mir-458) was associated with loss of tumor suppressive functions (143). In agreement with this, gga-miR-375 was found to be downregulated in the liver of ALV-J-infected chicken compared to non-infected animals. Overexpression of this miRNA led to a significant inhibition in the proliferative capacity of DF-1 chicken fibroblasts, likely *via* targeting and repressing yes-associated protein 1, cyclin E, and *Drosophila* inhibitor of apoptosis protein 1 mRNAs (144). Conversely, increased replication of ALV-J was associated with the upregulation of miR-23 in the spleen of ALV-J-infected chickens. This miRNA can target and suppress interferon regulatory factor 1, thus allowing enhanced virus replication (145). Similarly, Dai et al. found that miR-221 and miR-222, which were upregulated in the liver of ALV-J-infected chickens, can act as tumorigenic agents by targeting BCL-2 modifying factor (146). In another study, microarray analysis of liver tumors from ALV-J-infected chicken indicated that gga-miR-221, gga-miR-193a, gga-miR-193b, and gga-miR-125b were differentially expressed. Gene ontology and pathway analyses of these miRNAs indicated that ALV-J-triggered tumorigenesis may be, at least in part, due to these miRNAs targeting the MAPK and Wnt signaling pathways (147). Collectively, these data indicate that miRNAs form an integral part of the host response to ALV-J infection and can influence its pathogenesis by promoting or repressing tumor formation. They also suggest that modulating miRNA expression does have potential for interventional strategies against ALV-J infection.

### Infections in Laboratory Animal Models

#### Venezuelan Equine Encephalitis Virus

Venezuelan equine encephalitis virus (VEEV) is an equine disease that can be transmitted to humans *via* a mosquito vector and cause lethal inflammation of brain tissue. Mortality rates in equines have been estimated at 19–83% but are below 1% in humans (148). Reports suggest that miRNA expression is highly regulated upon VEEV infection of neurons and glial cells. Bhomia et al. (92) were the first to describe miRNA expression patterns in a mouse model of VEEV infection. Twenty-five miRNAs were upregulated, and 4 were downregulated in brain tissue collected after 48 and 72 h (Table S1 in Supplementary Material). Mmu-miR-155 showed >5-fold higher expression at both time points, suggesting that it might serve as an indicator of VEEV infection. These miRNAs may play important roles in modulating gene expression and neuronal degeneration in the brain following VEEV infection.

#### Rabies Virus

Rabies virus (RV) is a neurotropic virus that can infect the central nervous system (CNS) and lead to death in many cases of human infection. Canines constitute the main reservoir, but nearly all warm-blooded animals can contract the infection. The disease is transmissible from dogs to humans. Every year, it causes 55,000 human deaths worldwide (149). Thus, controlling the disease in domestic dogs has important implications for public health. In mice challenged with RV, a strong modulation in the expression of miRNA molecules was observed (Table 1), and the differentially expressed miRNAs are thought to be involved in several immune-associated signaling pathways (93). In another study, miR-133, which is specifically expressed in skeletal muscle, was predicted to bind to both N and G transcripts of RV (150). This might be a plausible explanation for the lengthy dormant state of this virus in skeletal muscle during the early phase of infection and before the continuation of its journey through the nervous system.

#### Prion Protein-Related Diseases

Prion proteins are the causative agents of bovine spongiform encephalopathy (BSE; “mad cow disease”) in cattle, feline spongiform encephalopathy in cats, scrapie in sheep, and Creutzfeldt–Jakob disease (CJD) in humans. This group of fatal neurodegenerative disorders is caused by an abnormally folded form of the naturally occurring cellular prion precursor protein, PrP^c^. Over the last 15 years, intense efforts have been undertaken following the appearance of a new prion disease that is transmissible to humans, variant Creutzfeldt–Jakob disease (VKJD). Ingestion of meat from BSE-infected cattle or scrapie-infected sheep is the source of infection (151). There is compelling evidence of a role played by miRNAs in the pathogenesis of the prion-related diseases. Brain of mice infected with mouse-adapted scrapie showed a unique expression pattern of 15 miRNAs that might act during prion-induced neurodegeneration. Most of them were upregulated more than 2.5-fold (Table 1). Among these, only miR-128 had previously been shown to be dysregulated in other neurodegenerative diseases, suggesting a pattern specific for these closely related diseases (94, 95). To determine whether miRNA dysfunction is involved in prion disease pathogenesis, the authors used a BSE-infected cynomolgus macaque model to confirm that miR-342-3p was upregulated in brain (152). Moreover, the authors confirmed that hsa-miR-342-3p was upregulated in brain samples of human type 1 and type 2 sporadic CJD, suggesting that miR-342-3p may be a biomarker of prionopathies in animals and humans. This also indicates that this miRNA might affect pathogenesis in different species. In another study, Taganov et al. proposed a role for miR-146a as a potent modulator of microglial function by controlling the activation state during prion-induced neurodegeneration (153). In general, miR-146a can directly downregulate the production of pro-inflammatory cytokines by acting as a negative-feedback effector of the NF-κB pathway (154). A coordinated dysregulation of miRNAs seen in prion diseases may be a response to the abnormal accumulation of PrP^Sc^, leading to signaling pathways that induce alterations in neurotransmitter receptors and protein degradation, resulting in the ultimate failure of neuronal function.

### Infections in Cellular Models

In addition to the above mentioned *in vivo* models, there are several examples of *in vitro* trials that have unraveled multiple roles of cellular miRNAs in different scenarios of infections with animal viral diseases of veterinary importance. One prominent example can be seen in the oncogenic miR-155 (155). miR-155 was initially identified as a gene that was activated by promoter insertion at a common retroviral integration locus in B-cell lymphomas called B-cell integration cluster (*BIC*) (156). B-cell lymphomas express greater levels of miR-155 and *BIC* transcripts than normal B cells as both of them are processed from the same primary transcript (157). By generating *bic*/miR-155-deficient mice, Rodriguez et al. provided evidence that absence of *BIC*/miR-155 resulted in lung fibrosis mimicking the picture of complicated autoimmune diseases, reduced amount of immunoglobulin M, and low levels of *IL-2* and *IFN-*γ, suggesting a key role for miR-155 in regulating immune responses (158). Extensive studies have been performed thereafter to illustrate miR-155 roles in tumor viruses affecting animals. One example is the reticuloendotheliosis virus strain T (REV-T). Infection of chicken embryo fibroblasts and REV-T-induced B-cell lymphomas demonstrated elevated miR-155 levels (159). Further functional studies revealed that miR-155 can target JARID2 mRNA (a cell cycle regulator) causing its downregulation, reflecting the role of miR-155 in promoting cell survival. Using a comparable *in vitro* approach, miR-181a was linked to MDV-induced lymphoma. In this regard, transfecting miR-181a mimic into MYBL1 cells, a Marek’s disease lymphoma cell line, resulted in the reduction of v-myb myeloblastosis viral oncogene homolog-like 1 protein. The study also showed an inhibitory effect of gga-miR-181a on cell proliferation (160). A similar role was reported for miR-26a in MDV infection, where it inhibited lymphoma cell proliferation by targeting *NEK6*, thus playing a role as a tumor suppressor (133). Transfection of a lentiviral vector expressing miR-21 into DF-1 cells has been demonstrated to inhibit IBDV replication *via* targeting the VP1 gene of this virus (161). Using a luciferase reporter assay, Wang et al. proved that chicken miR-1650 can bind the 5′-UTR of the ALV-J Gag mRNA, therefore influencing virus replication (162). In bovine medicine, bovine viral diarrhea is a significant disease of cattle, which is still endemic in many parts of the world (163). The role of miRNA in bovine viral diarrhea infection has been illuminated in two recent studies. In the first, the authors reported that miR-29b can bind to the 3′-UTR of two key apoptosis-related genes, caspase-7 and nuclear apoptosis-inducing factor 1, causing their downregulation in Madin–Darby bovine kidney (MDBK) cells. They also showed that miR-29b could target the viral envelope glycoprotein with subsequent suppression of viral replication (164). In the second study, the same researchers proved that lentivirus-mediated overexpression of the miR-29b precursor reduced replication of bovine viral diarrhea virus in MDBK cells by downregulating the expression of ATG14 and ATG9A, two important autophagy-associated proteins (165). These studies emphasize that miR-29b can modulate the pathogenesis of bovine viral diarrhea virus using different mechanisms. Along the same lines, PK-15 cells were used to screen for differentially expressed miRNAs during foot-and-mouth disease virus (FMDV) infection (166). The analysis revealed that 172 known miRNAs and 72 putative miRNAs were differentially expressed, the majority of which were downregulated. Bioinformatics analyses predicted that the target mRNA genes of these differentially expressed miRNAs were involved in pathways such as cytokine receptor signaling, NOD-like receptor signaling, and toll-like receptor signaling. Another example from the swine field can be seen in porcine reproductive and respiratory syndrome virus (PRRSV) infection, which is a main cause of late abortion and respiratory diseases in pigs (167). Several *in vitro* trials were performed to understand the role of miRNAs in the pathogenesis of PRRSV infection. Recently, it was found that PRRSV infection triggered miR-24-3p expression that downregulated heme oxygenase-1 mRNA, which was associated with an overall increase in PRRSV replication in the MARC-145 cell line (168). Other reports demonstrated the ability of some miRNAs to restrict PRRSV replication. For instance, miR-26a acts by enhancing type I IFN-signaling pathways and augmenting the production of IFN-stimulated genes, thus blocking virus replication (169). A virus inhibitory effect is mediated also by miR-506, which suppresses the mRNA encoding CD151, an important receptor of PRRSV (170). In the MARC-145 cell line and primary porcine alveolar macrophages, PRRSV induced downregulation of miR-125b as a way to enhance its replication. This miRNA was found to block the NF-κB pathway, which is essential for PRRSV replication (171). Profiling studies of PRRSV-infected porcine alveolar macrophages revealed altered expression of 40 miRNAs, including miR-30a-3p, miR-132, miR-27b*, miR-29b, miR-146a, and miR-9-2, of which miR-147 could be shown to inhibit virus replication (172). Swine testis cells infected with transmissible gastroenteritis virus (TGEV) uniquely expressed 59 miRNAs (15 upregulated and 44 downregulated). The authors argued that these miRNAs are involved in targeting host signaling pathways, including metabolic and immune-related pathways (173). A similar profiling study in TGEV-infected PK-15 cells demonstrated that increased expression of miR-4331 can suppress the transcription of TGEV gene 7 *via* targeting cellular cell division cycle-associated protein 7 (174). Recently, overexpression and silencing studies revealed that miR-27b is an important suppressor of apoptosis triggered by TGEV, possibly by targeting the runt-related transcription factor 1 gene. The authors concluded that the virus can induce apoptosis by downregulating this miRNA in the infected cells (175). Targeting elements of a viral genome is an intriguing way by which miRNAs may interfere with viral infections. For instance, miR-181c was found to bind a highly conserved region in the PRRSV genome, causing specific and dose-dependent reduction in virus titers in Marc-145 cells, implicating miR-181c in a pathogen-specific host response to PRRSV infection and supporting the idea of using miRNAs in the control of PRRSV infection (176). Similar inhibitory mechanisms (targeting MDV in this case) have been reported for miR-23, miR-378, and miR-505 (133). Eastern equine encephalitis virus (EEEV) constitutes another example where a host miRNA modulates viral activity. miR-142-3p inhibits EEEV replication in myeloid cells by binding to its 3′-UTR, which leads to a reduced immune response accompanied by exacerbated disease manifestations in the CNS. An intriguing opposite effect is seen in the mosquito vector in that miR-142-3p binding sites in the 3′-UTR are required for efficient viral replication, which would augment transmission to the mammalian hosts (177).

Taken together, the results of the studies presented in this section provide additional evidence for the importance of host miRNAs in the pathogenesis of viral infectious diseases of animals and provide encouraging preliminary data for the use of small non-coding RNAs targeting viral genomes as antiviral interventional strategies.

## Perspectives

Many miRNAs have been identified that may affect pathogenesis and outcome of viral infectious diseases of veterinary importance. Nevertheless, many open questions remain. Inter-host differences in susceptibility to a given viral infection are often due to differences in mRNA and, subsequently, protein expression, and the intriguing role of miRNAs in regulating these differences certainly requires further investigations. In this regard, we propose that the study of highly pathogenic avian influenza (HPAI) infections in different avian and mammalian hosts would constitute a promising model system of high relevance to veterinary practice. While infection is asymptomatic in waterfowl, humans and chicken are more susceptible and develop a concurrent strong inflammatory response and high tissue cytokine levels (178). Also, pathogenicity of HPAI H5N1 viruses varies among various breeds of ducks (179). In the same context and as a deviation from the normal ecology of the virus, some recent isolates of H5N1 proved to be lethal for waterfowl species, including ducks (180). Given these observations, comparing the expression patterns of host-encoded miRNAs in response to HPAI in different hosts, organs, and disease states might explain their difference in susceptibility to HPAI infection and help to identify additional elements of the host response that affect disease severity. One research area of great scientific interest and of clinical importance as well is to investigate the role of miRNAs in the development of viral and bacterial coinfections, such as IAV/*S. pneumoniae* in humans and IAV/*Escherichia coli* in chicken and ducks (181), as the role of host miRNAs in modulating potential synergies between two pathogens may be substantial. Human populations experienced the emergence of zoonotic diseases, in particular those caused by viruses that cause varying numbers of human fatalities. It is important to invest more efforts to delineate the role of miRNAs that are associated with these zoonotic viral diseases, especially when considering their cross-species conservation. This will improve our understanding of the complex nature of zoonotic pathogens as well as their potential to further establish interhuman transmission.

## Conclusion

**Figure 4:**
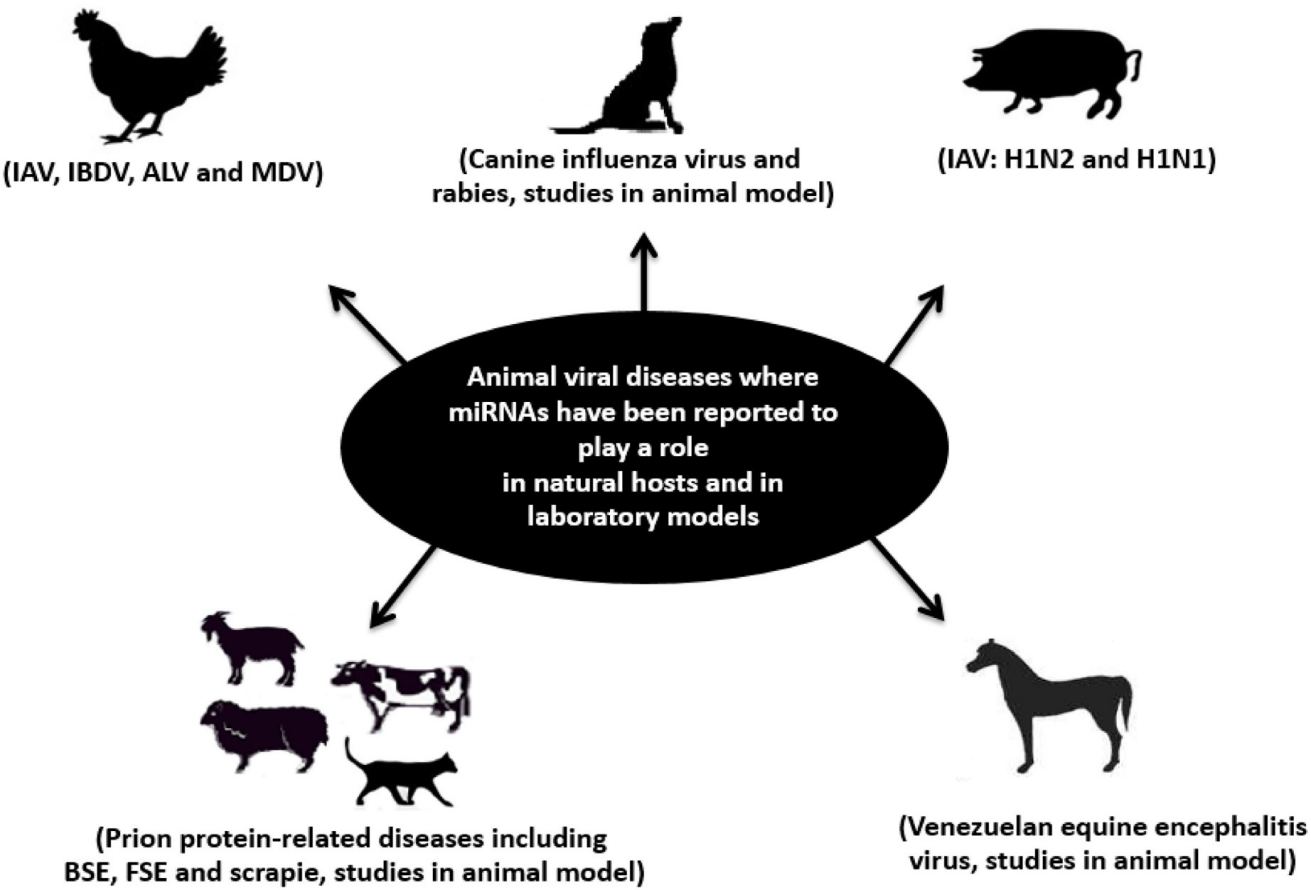
**Schematic representation of infectious viral diseases of farm and pet animals (in natural hosts and in laboratory models) where miRNAs have been reported to play a role**. Examples of these diseases are bovine spongiform encephalopathy (BSE); feline spongiform encephalopathy (FSE); rabies; influenza A virus (IAV); infectious bursal disease virus (IBDV); Marek’s disease virus (MDV); avian leukosis virus (ALV); scrapie; and Venezuelan equine encephalitis (VEE). Adapted from Ref. (182).

The discovery of small non-coding RNAs was a turning point in biology. The role of miRNAs has grown with an unprecedented speed from research on worms to a wide variety of physiological and pathological processes in humans and animals. With genome-wide profiling techniques and the tools of bioinformatics, considerable information about miRNAs and their role in animal viral diseases is now available. The current experimental data on the role of miRNAs in host–virus interaction upon infection of a natural host, in laboratory models and in cell-based systems, indicate that miRNAs can contribute to both pathogenesis and clinical outcome of many diseases affecting animal populations on the individual and farm level (Figure 4). However, a general conclusion on the role of miRNAs cannot be drawn yet. One possibility is that they favor the host as part of the antiviral response. This could occur in two ways. First, miRNAs could silence viral transcripts through sequence-specific binding, and thus protein expression. Second, they could indirectly modulate host transcripts in a way that creates a less favorable condition for virus propagation and survival. *Vice versa*, host miRNAs may be beneficial for the viral pathogens if they tend to increase their replication or survival. Finally, they can be beneficial for both sides as in the case of latent viruses. The role of miRNAs in veterinary medicine is receiving more and more attention. Considering the current efforts to include more hosts of veterinary importance in online miRNA databases, as well the increased availability of high-throughput profiling approaches and functional studies, our understanding of the roles of miRNAs in viral infections of veterinary importance will likely continue to improve and lead to tangible clinical applications in the foreseeable future.

## Author Note

This review paper is part of the PhD thesis of Mohamed Samir Ahmed, completed at the University of Veterinary Medicine (Stiftung Tierärztliche Hochschule), Hannover, Germany.

## Author Contributions

MA conceived the project, did the literature search, wrote the initial draft of the manuscript, and prepared the figures and tables. LV participated in the bioinformatics analyses and edited the manuscript. FP oversaw the project, edited the manuscript, and takes responsibility for the integrity of the data.

## Conflict of Interest Statement

The authors declare that the research was conducted in the absence of any commercial or financial relationships that could be construed as a potential conflict of interest.

## Abbreviations

3′-UTR, 3′-untranslated region; ALV, avian leucosis virus; BIC, B-cell integration cluster; BSE, bovine spongiform encephalopathy; CJD, Creutzfeldt–Jakob disease; CNS, central nervous system; DGCR8, DiGeorge syndrome critical region 8; DIANA, DNA intelligent analysis; GaHV-2, Gallid herpesvirus 2; HPAI, highly pathogenic avian influenza; IBDV, infectious bursal disease virus; IAV, influenza A virus; lncRNAs, long non-coding RNAs; MDV, Marek’s disease virus; miRNAs, microRNAs; NLRP3, NOD-like receptor P3; nts, nucleotides; PRRSV, porcine reproductive and respiratory syndrome virus; pre-miRNA, precursor miRNA; pri-miRNA, primary miRNA; RISC, RNA-induced silencing complex; RNA seq, RNA sequencing; RT-qPCR, reverse transcription quantitative real-time PCR; RV, rabies virus; REV-T, reticuloendotheliosis virus strain T; SDN, small RNA degrading nucleases; snoRNA, small nucleolar RNA; SOCS, suppressor of cytokine signaling; TGEV, transmissible gastroenteritis virus; TRBP, TAR RNA binding protein; VEEV, Venezuelan equine encephalitis virus; VCJD, variant Creutzfeldt–Jakob disease.
